# A Heterologous Viral Protein Scaffold for Chimeric Antigen Design: An Example PCV2 Virus Vaccine Candidate

**DOI:** 10.3390/v12040385

**Published:** 2020-03-31

**Authors:** Emilio Lamazares, Fernando Gutiérrez, Angela Hidalgo, Nicolas A. Gutiérrez, Felipe I. Espinoza, Oliberto Sánchez, Marcelo Cortez-San Martín, Carolina Mascayano, Javier González, José Saavedra, Claudia Altamirano, Manuel Mansur, Álvaro Ruiz, Jorge R. Toledo

**Affiliations:** 1 Biotechnology and Biopharmaceutical Laboratory, Departamento de Fisiopatología; Facultad de Ciencias Biológicas, Universidad de Concepción, Víctor Lamas 1290, P.O. Box 160-C, Concepción 4079386, Chile; elamazares@udec.cl (E.L.); felipeiespinozar@gmail.com (F.I.E.);; 2 Molecular Virology and Pathogen Control Laboratory, Departamento de Biología, Facultad de Química y Biología, Universidad de Santiago de Chile (USACH), Alameda 3363, Correo 40, Casilla 33, Santiago 9170022, Chile; 3 Departamento de Ciencias del Ambiente, Facultad de Química y Biología, Universidad de Santiago de Chile (USACH), Alameda 3363, Correo 40, Casilla 33, Santiago 9170022, Chile; 4 Natural History Museum of Potsdam, 14467 Potsdam, Germany; 5 Laboratorio of Cultivos Celulares, Escuela de Ingeniería Bioquímica, Pontificia Universidad Católica de Valparaíso, Ave. Brasil 2085, Valparaíso 2362803, Chile; 6 Moderna Therapeutics 100 Upland Rd., Norwood, MA 02062, USA; 7 Pathology and Preventive Medicine Department, School of Veterinary Sciences, Universidad de Concepción, Ave. Vicente Méndez 595, Chillan 3812120, Chile

**Keywords:** PCV2 virus, recombinant antigens production, vaccines, biotechnology strategies

## Abstract

Recombinant vaccines have low-cost manufacturing, regulatory requirements, and reduced side effects compared to attenuated or inactivated vaccines. In the porcine industry, post-weaning multisystemic disease syndrome generates economic losses, characterized by progressive weight loss and weakness in piglets, and it is caused by porcine circovirus type 2 (PCV2). We designed a chimeric antigen (Qm1) to assemble the main exposed epitopes of the Cap-PCV2 protein on the capsid protein of the tobacco necrosis virus (TNV). This design was based on the Cap-N-terminal of an isolated PCV2 virus obtained in Chile. The virus was characterized, and the sequence was clustered within the PCV2 genotype b clade. This chimeric protein was expressed as inclusion bodies in both monomeric and multimeric forms, suggesting a high-molecular-weight aggregate formation. Pigs immunized with Qm1 elicited a strong and specific antibody response, which reduced the viral loads after the PCV2 challenge. In conclusion, the implemented design allowed for the generation of an effective vaccine candidate. Our proposal could be used to express the domains or fragments of antigenic proteins, whose structural complexity does not allow for low-cost production in *Escherichia coli*. Hence, other antigen domains could be integrated into the TNV backbone for suitable antigenicity and immunogenicity. This work represents new biotechnological strategies, with a reduction in the costs associated with vaccine development.

## 1. Introduction

Recombinant protein production for diagnostic or therapeutic applications, in humans or animals, has increased over recent decades [1]. The worldwide market volume of the pharmaceutical industry in 2017 was US$187 billion, while the market for recombinant proteins accounted for approximately one-third of those sales [2,3]. Currently, there are over 400 marketed recombinant products and another 1300 are under development and clinical trials. Most of these were obtained through biotechnology processes in organisms, such as bacteria, yeasts, and insect and mammalian cells [4]. Recombinant protein-based vaccines constitute an expanding market, due to the cost-effective manufacturing with the advantage of reduced side effects and regulatory requirements when compared with attenuated or inactivated vaccines [5,6].

Post-weaning multisystemic disease syndrome (PMWS) is characterized by leading to a series of illnesses, such as reproductive disorders, enteric and respiratory diseases, adenopathies, progressive weight loss, and weakness in piglets and growing pigs [7]. The deterioration in the pig farms that suffer PMWS triggers great economic losses in the livestock industry [8,9], with the porcine circovirus type 2 (PCV2) as the etiological agent of this syndrome [10]. The PMWS disease develops only in animals coinfected with PCV2 and other pathogens, such as *Mycoplasma hyopneumoniae*, *Actinobacillus pleuropneumoniae*, *Salmonella* spp., classical swine fever virus (CSFv), porcine reproductive and respiratory syndrome, Aujeszky’s disease, porcine parvovirus (PPV), porcine influenza virus (PIV), and African swine fever virus (ASFV) [11,12]. As the pathologies generated by this syndrome are diverse, these have been renamed as porcine circovirus associated disease (PCVAD) [13,14].

PCV2 belongs to the Circovirus genus of the Circoviridae family, conformed by PCV1, PCV2, and PCV3 [15], and has the greatest geographical distribution [16]. PCV2 infects the cells of the immune system, such as the dendritic cells, monocytes, and macrophages [17,18], negatively modulating the regulatory cytokine pattern of the immune response, which causes lymphocyte depletion and immunodeficiency in infected pigs [19,20,21]. This immunodeficiency allows the infection with other concomitant pathogens, which provokes the death of infected animals [22].

This pathogen consists of a non-enveloped circular single-stranded DNA virus, with a length of 1.7 kbp [23]. The capsid is composed of a single structural protein (Cap), which is expressed under the open reading frame 2 (ORF2) with a molecular weight of 27.8 kDa [24], and is considered the main immunogenic protein of this virus [25]. Currently, the vaccines to prevent PMWS, are grouped into three types: inactivated, chimeric, and subunit vaccines [26]. Specifically, subunit vaccines against PCV2 are based mainly on virus-like particles (VLPs), expressed and assembled “in vitro” in insect cell cultures; however, this expression system is associated with a high production cost for veterinary purposes [27].

Although currently marketed vaccines certainly protect pigs from the clinical status of PMWS, these do not eliminate subclinical infections, thus maintaining the virus in porcine livestock. This problem, in turn, demands constant vaccination rounds for each new piglet herd, which results in increased costs for the porcine industry [28,29]. More recently, experimental vaccines based on the chimeric viruses PCV1 and PCV2 were formulated in chimeric porcine circovirus-Cap proteins, where small Cap loops were replaced with other epitopes of interest. These chimeric proteins were expressed in *Escherichia coli (E. coli)*, preserving the structure of the VLP [30,31,32], and this approach was demonstrated as an alternative against PCV2 infection.

As most of the viral genetic and protein sequences of the pathogen are patent protected, which hinders the registration of a new commercial vaccine, we decided to explore a new possibility, by developing a chimeric vaccine using a heterologous protein scaffold “decorated” with pathogenic epitopes.

In the present work, we propose the development of a new antigenic variant for a Cap-tobacco necrosis virus (TNV) derived vaccine decorated with PCV2 epitopes. This is based on a Cap-N-terminal of a Chilean isolate PCV2 virus, combined with other reported epitopes for PCV2 consensus sequences, which were assembled over structural regions of the capsid-TNV virus, identified as Cap-PCV2 homologous with a similar structural conformation. This new chimeric variant was expressed in *E. coli*, a simple, cheap, and efficient expression system, mainly as inclusion bodies. Additionally, the protein oligomer status observed by electrophoretic methods suggests high-molecular-weight protein aggregates. This antigen was formulated as a water-in-oil emulsion for a pig vaccination assay. The immunized animals elicited a highly specific antibody response against the Cap-PCV2 chimeric antigen, which reduced the viral load after challenge.

The present approach represents combined molecular and biotechnology tools for the design and manufacturing of new viral chimeric antigens derived from complex proteins expressed in a simple and economical system, such as *E. coli*.

## 2. Materials and Methods

### 2.1. Plasmid, Gene Cloning, and Strain

The synthetic gene of chimera 1 (Qm1) GenScript (Nanjing, China) was cloned into X*ho*I-N*de*I sites of the vector pET-22b (+). *E. coli* strain SHuffle^®^ T7 Express (New England Biolabs, Ipswich, MA, USA) was used as the expression strain.

### 2.2. Design of the Recombinant Chimeric Antigen (Qm1)

The sequence of a viral isolate obtained during PCV2 outbreaks, associated with high pig mortalities in Chile in 2007, was analyzed. The viral isolate was genetically characterized and the sequence of the gene coding for the capsid protein was named “PCV2 Chile Rancagua2007 Cap gene” (Cap gene).

#### 2.2.1. Protein Sequence Similarity Analyses

We analyzed the amino acid sequence similarity of the PCV2 capsid gene by using classical multidimensional scaling (CMDS) plots. Distance matrices were calculated from the single gene (capsid) alignment using the program Protdist included in the package Phylip v. 3.6 [33,34]. Distance estimation was based on the model of amino acid substitution DCMut [35], a version of the PAM model of Margaret Dayhoff [34]. CMDS was performed with the function ‘cmdscale’ available in R v. 3.3.2 [36].

#### 2.2.2. Phylogenetic Analysis

Discarding predicted recombinant sequences and following Franzo et al. (2015) [37], we retrieved, from the Genbank database, a total of 902 reference sequences of porcine circovirus type 2 (PCV2), family Circoviridae, genus Circovirus. Only sequences assigned to replicase (rep) and capsid (cap) genes were parsed later into two final alignments. These alignments contained four genotypes (a, b, c, and d) and a total of 846 and 587 sequences (including putative genes) for the capsid and concatenated capsid and replicase genes, respectively (Supplementary Material, Table S1).

The PCV2 sequence found in samples from Chile (“Rancangua 2007”) was aligned to the GenBank sequences by using Muscle v. 3.8.31 [38] and the resulting alignment was then checked by eye with BioEdit v. 7.0.5.3 [39]. We explored the model of sequence evolution and partitioning schemes that fit the data best with PartitionFinder 2.1.1 [40] and the “--raxml option” [41]. In these analyses, we made use of the corrected Akaike information criterion (AICc), linked branch lengths, and greedy search [40]. For the concatenated data set (rep + cap genes), we set six data blocks for partitioning schemes analyses, i.e., the first, second, and third codon positions of the two protein-coding genes (rep and cap) found to be in the genome of the PCV2. Similarly, we set three data blocks for the single gene alignment using the first, second, and third codon positions of the capsid gene.

Phylogenetic analyses were run separately for the capsid DNA sequences and concatenated capsid + replication DNA sequences. We reconstructed phylogenetic trees using maximum likelihood (ML) in RAxML v. 8.2.12 [42]. In the ML analyses, we initially searched the best tree via 100 ML searches on distinct starting trees, assigning the GTR + GAMMA model to the alignment partitions found with PartitionFinder. Finally, the robustness of each node was assessed by 1000 bootstrap replicates and the trees were visualized with FigTree v 1.4.0 [43].

#### 2.2.3. Structural Homologs for PCV2 Capsid Protein

The structural homologs to the sequence reported for Cap (PDB ID: 3r0r) were made through the TopSearch server and the alignment between the structural and Cap homologs was performed in 2013 using the TopMatch server. Finally, three structural homologs were obtained as tobacco necrosis virus (TNV), human rhinovirus 16 (HRV16), and rice yellow mottle virus (RYMV) with PDB IDs: 1c8n, 1aym, and 1f2n, respectively. Finally, the structural homologs analysis showed that TNV was the most similar structure compared with the template as demonstrated in the Supplementary Material, Table S2.

To determine the secondary structure conservation between the Cap protein (PDB ID: 3r0r) and the three proteins found, a sequence alignment was performed using the structural alignment with the VAST server (https://structure.ncbi.nlm.nih.gov/Structure/VAST/vast.shtml), using these results, and the corresponding alignments, we designed the chimeric proteins (Figure S2).

#### 2.2.4. Recombinant Chimeric Antigen Design

In order to design the chimeric protein candidate to maintain the protein structure of the PCV2 Cap protein and the immunogenic epitopes using the TNV protein backbone, the VAST alignment of Figure S2 was used. The five most important epitopes were obtained from the previous study published by Shang et al. in 2009 and these sequences were replaced over the TNV protein backbone regarding the secondary structure. The chimeric amino acid sequence is called Qm1 [44].

#### 2.2.5. Protein Modeling of Chimeras

The similarity of the amino acid sequence between the Qm1 protein and sequence from the resolved cap structure (3r0r, GI340707970), was analyzed using the Clustal Omega program with the default comparison options (Figure 4A). 

The comparative model of the antigenic variant of PCV2 was obtained using the information of the secondary structure alignment that was previously made between the Cap sequence reported for PCV2 and the structural counterpart. The antigenic variant was constructed with the MODELLER 9.11 program, using the three homologs (1c8n, 1aym, and 1f2n) as templates. One hundred models were obtained for each chimeric structure (Qm1, Qm2, and Qm3), and these were ordered using the criteria of both energy stability and structural quality. Each best model was selected, and the structures were optimized by means of energy minimizations, using the Charmm33b1 force field to eliminate the steric hindrances produced between the atoms, due to the positioning of each of the side chains of the amino acid residues that make up the protein. Next, the visual molecular dynamics visualization program (VMD) was used to analyze the antigenic variant for each of the built chimera.

Finally, the model selected and used in this study was called Chimera1 (Qm1). The epitopes of the amino-terminal of the isolate obtained in Rancagua2007 were added. The nucleotide and amino acid sequences of Chimera1 were called “PCV2 Cap Chimera1 gene” and “PCV2 Cap Chimera1 protein”, respectively (Figure 4B).

### 2.3. Bacterial Transformation and Small-Scale Expression Analysis

The recombinant Qm1 expression was assessed in the SHuffle^®^ T7 Express *E. coli* strain and heat-shock transformed with pET22-Qm1. Six of the colonies were selected after overnight growth on LB medium (Luria–Bertani, Liofilchem (TE), Italy) with 100 µg/mL ampicillin (USBiological, USA) (LBA) at 37 °C for expression analysis. The culture was carried out in 250 mL culture flasks with 50 mL of LBA medium at 37 °C and 200 rpm. Induction with 0.5 mM isopropyl β-D1-thiogalactopyranoside (IPTG) from Santa Cruz Biotechnology (Dallas, Texas, USA) was performed when the cell concentration reached at 0.6–0.8 OD_600_. The Qm1 expression was verified by sodium dodecyl sulfate-polyacrylamide gel electrophoresis (SDS-PAGE) and Western blotting.

### 2.4. SDS-PAGE and Western Blotting

For SDS-PAGE and Western blotting analysis, after 6 h, the induced cells were resuspended in Laemmli buffer and heated at 95 °C for 10 min. SDS-PAGE analysis was performed in 12.5% (*m*/*v*) polyacrylamide gels [45]. For Western blotting analysis, the proteins were transferred onto a nitrocellulose membrane (Schleicher and Schuell, Dassel, Germany) using a semidry TransBlot-Turbo electroblotter (Bio-Rad, Hercules, CA, USA). The membrane was incubated for 2 h at room temperature with a mouse anti-His-tag monoclonal antibody from Clontech Laboratories (Mountain Views, CA, USA) or a rabbit polyclonal anti-Cap antibody. Secondary antibody incubation was then made for 1 h at room temperature with a goat Alexa fluor^®^ 680 labeled anti-mouse (or anti-rabbit) polyclonal antibody from Jackson ImmunoResearch (West Grove, PA, USA). The infrared signals were measured using the Odyssey System from LI-COR Biosciences (Lincoln, NE, USA).

### 2.5. Release of the Polyclonal Antibody against Proteins of the SHuffle ^®^ T7 E. coli Strain

One gram of cells of the untransformed SHuffle ^®^ T7 Express *E. coli* strain was suspended in 40 mL of PBS (phosphate-buffered saline). The cell rupture was carried out using an Emulsiflex C-5 homogenizer from Avestin (Ottawa, ON, Canada) carrying out eight passes of the sample. Subsequently, 10 mL of the disrupted sample was centrifuged at 4470 g for 20 min. The rupture supernatant was separated from the pellet and precipitated with 76% (*m*/*v*) Trichloroacetic acid (TCA) [46]. The commercial anti-Cap polyclonal antibody was prepared according to the manufacturer’s instructions using a 1:400 dilution in 2% (*m*/*v*) skimmed milk dissolved in Tris-buffered saline (TBS). Then, 20 mL of the antibody solution was taken and homogenized with the disrupted cells, and the culture supernatants were processed with TCA and vortexed for 1 min. The sample was incubated at 4 °C with stirring overnight. Subsequently, the sample was centrifuged at 4470× *g* for 30 min, discarding the precipitate. The supernatant was separated and the released serum against SHuffle ^®^ T7 Express proteins was stored in 1 mL aliquots at −20 °C.

### 2.6. Batch Fermentation Conditions

The cell cultures were performed in a 5 L Winpact bioreactor (Major Science, Saratoga, CA, USA). To prepare the inoculum, the strain was first streaked on an LBA plate and incubated at 37 °C for 12 h. From the fresh plate, two 1 L flasks with 200 mL of LBA medium were inoculated and incubated at 37 °C for 12 h. Fermentation was performed at 37 °C. The pH was kept constant at 7.0 by the controlled addition of 25% (*m*/*v*) NH_4_OH and 3M H_3_PO_4_, and the dissolved oxygen was kept constant at 10% by increasing the agitation between 200 and 300 rpm in a total volume of 5 L. Once the culture reached an OD_600_ of 0.6–0.8, expression was induced by the addition of 0.5 mM IPTG. After 6 h of the induction period, the culture was centrifuged at 11,000× *g* for 10 min, and the wet biomass was weighed and stored at −20 °C.

### 2.7. Biomass Disruption and Protein Detection

The biomass was resuspended by weighing 1 g in 40 mL of PBS buffer pH 7.4. The cells were disrupted by passing eight times through a high-pressure homogenizer cell at 48 MPa (480 bar) using an EmulsiFlex C-5 apparatus (Avestin, Inc). The homogenized product was centrifuged at 4470× *g* for 20 min, and the cell disruption supernatant was separated from the pellet. The latter was resuspended in the same volume obtained from the rupture supernatant using PBS buffer pH 7.4, and both samples were stored at -20 °C. The Qm1 expression was detected by SDS-PAGE with the denaturing condition and by Western blotting using an anti-poly-histidine monoclonal antibody and anti-Cap polyclonal antibody.

### 2.8. Qm1 Purification by Immobilized Metal Affinity Chromatography (IMAC)

For the Qm1 purification from SHuffle^®^ T7 Express *E. coli*, the cells were resuspended in PBS buffer and lysed by a mechanical disruption in an EmulsiFlex C-5 High-pressure homogenizer from Avestin (Ottawa, ON, Canada) and centrifuged at 17,500× *g* for 10 min at 4 °C to separate the pellet of the soluble fraction. The soluble fraction was loaded onto a Ni^2+^ charged column IMAC SepharoseTM 6 Fast Flow, connected to an ÄKTA prime plus system, both from GE Healthcare (Chicago, IL, USA). The column was equilibrated with five column volumes (CVs) of PBS buffer, then the sample was loaded and the flow-through was collected for analysis. The elution was made using Imidazole in PBS buffer at increasing concentrations: 25, 50, 150, and 400 mM (Supplementary Material, Figure S3). The purified Qm1 protein was quantified by SDS-PAGE by densitometry using bovine serum albumin as an external standard. The images were captured and processed using the LI-COR Odyssey imaging system program Image Studio v 3.1 (LI-COR, USA).

### 2.9. Formulation and Dose Distribution

An oil-based vaccine formulation was made with the recombinant chimeric antigen Qm1 as an active principle. We used a concentration of 100 μg of Qm1 antigen for every 2 mL of the final formulation, which corresponds to the dose per pig. The Montanide ISA 50 V2 adjuvant from SEPPIC (Paris, France) was used in a ratio of 20:80 oil in water. The sample was homogenized using Ultra-Turrax equipment from Janke&Kunkel (Staufen, Germany). The homogenization cycles consisted of 2 min of mixing at 10,000 rpm and 30 s of rest until no separation of the phases was observed. The stability of the emulsion was evaluated for at least five days of post-emulsion and its safety was tested in mice in compliance with the national guidelines (Chilean Law N° 20.380, 2009) and the authorization of the Ethical Committee of the Universidad de Concepción. The formulation remained stable without inducing reactogenicity or adverse effects in the inoculated mice. It was kept at 4 °C for two weeks, until use.

### 2.10. Animal Trials

#### 2.10.1. Safety Evaluation Test

The safety evaluation test was performed using 10 pigs of approximately three months of age. Five animals were immunized with 100 µg of Qm1, while the other five received a higher dose (300 µg of Qm1). Deep intramuscular immunization was performed in the neck. The inoculation area, the rectal temperature, the appetite, and the group behavior of the animals were observed daily for seven days to determine any change or evidence of reactogenicity or redness in the injection area.

#### 2.10.2. Immunization Assay

The pig studies were done in compliance with the national guidelines (Chilean Law Nº 20.380, 2009) and the authorization of the Ethical Committee of the Universidad de Concepción. The immunological response of the recombinant protein was evaluated in healthy piglets that were three weeks old. Two experimental groups of eight pigs each were immunized by intramuscular injection with 100 μg of chimeric antigen 1 (Qm1) and saline solution (PBS 1X) as a negative control. Montanide ISA 50 V2 (Seppic, France) was used as an adjuvant in an antigen:adjuvant ratio of 60:40. The immunization schedule was performed with a first immunization (T = 0), a booster with the same amount of protein (T = 20), and a challenge test (T = 35). Blood samples were extracted every 10 days until day 60 of the assay.

#### 2.10.3. Challenge Test in Immunized Pigs with Recombinant Chimeric Antigens

Protection against pathogenic infection with porcine circovirus type 2 (PCV2) was performed in all experimental groups after 35 days of prime immunization. One dose of 1 × 10^7^ PCV2 viral particles was administered by the nasal route. The monitoring of animals during the challenge test was performed daily, based on behavior and clinical signs. After 25 days of the administration, blood samples were extracted to quantify the viral titer in serum.

All trials involving pigs were in accordance with the guidelines and recommendations of the NIH Guide for the Care and Use of Laboratory Animals (Eighth edition) and followed the policies indicated in the Chilean Biosafety Manual of CONICYT (National Agency for Science and Technology). The experimental protocols were drafted by the authors and approved by the Institutional Ethics Committee. In all cases, the supervision of veterinary authorities from the School of Veterinary Sciences, Universidad de Concepción, Chile, was guaranteed. The euthanized animals were handled humanly to avoid suffering.

#### 2.10.4. Immune Enzymatic Assay for Immune Response Evaluation

Flat-bottom 96-well ELISA plates (Nunc MaxiSorp™) were coated with 50 µg per well of purified Qm1 and after 16 h were diluted with coating buffer. Independent triplicate dilutions of each test animal serum were analyzed. Diluted pig serum was added to antigen-coated wells (100 μL/well) as the primary antibody and then, the plates were incubated with 100 μL/well of the appropriate secondary rabbit antibody and conjugated with peroxidase and 0.1 μg/well of goat anti-pig IgG polyclonal antibody H&L horseradish peroxidase labeled, from Abcam (Cambridge, USA). We added the substrate (0.1 mg/well of o-phenylenediamine dihydrochloride (OPD) from Sigma (St. Louis, MO, USA), diluted with coating buffer, and the absorbance at 492 nm was read using a Synergy™ HTX Multi-Mode Microplate Reader (BioTek). The titer was determined using the pre-immune serum absorbance value multiplied by two as the cut-off, using serum sample two-fold serial dilutions (from 1:500 to 1:64,000), and assigning the titer value as indicative of the last dilution in which the antibody was detected.

#### 2.10.5. Real-Time PCR for Viral Titer Analysis

DNA was purified from the serum sample of day 60 using the QIAamp DNA Mini kit from Qiagen (Hilden, Germany) according to the manufacturer’s instructions. The reaction was performed with the SensiMixTM SYBR Hi-ROX kit from Meridian Bioscience (Cincinnati, OH, USA), using the MX3000P equipment from Agilent Technologies (Santa Clara, CA, USA). The qPCR was performed using the DNA as template and water as a negative control, and the primers used were 5′-TGGCCCGCAGTATTCTGATT -3′ (forward) and 5′-CAGCTGGGACAGCAGTTGAG -3′ (reverse). The copy number was compared to the standard curve, and the values were indicative of copies of viral DNA per mL.

## 3. Results

### 3.1. Similarity Analysis of Sequences of PCV2 Isolates Worldwide

We used a viral isolate sequence representative of the Chilean strain, obtained from an outbreak of PCV2 that occurred in Rancagua city in 2007, for the bioinformatic design of the PCV2 antigenic variant. The Cap nucleotide sequence or Cap protein sequence were named as the “PCV2 Chile Rancagua2007 Cap gene” (Cap gene) or “PCV2 Chile Rancagua2007 Cap protein” (Cap protein) (Supplementary Material, Figure S1). The multiple sequence alignment showed that PCV2 Chile Rancagua2007 Cap protein has a 90% identity with respect to the reported Cap sequences (data not shown). However, it maintains a 100% identity in the amino-terminal region with the sequence of other isolates “ABW72700Chile2007”, as reported in Chile (data not shown).

### 3.2. Phylogenetic and Classical Multidimensional Scaling Analysis

**Figure 1:**
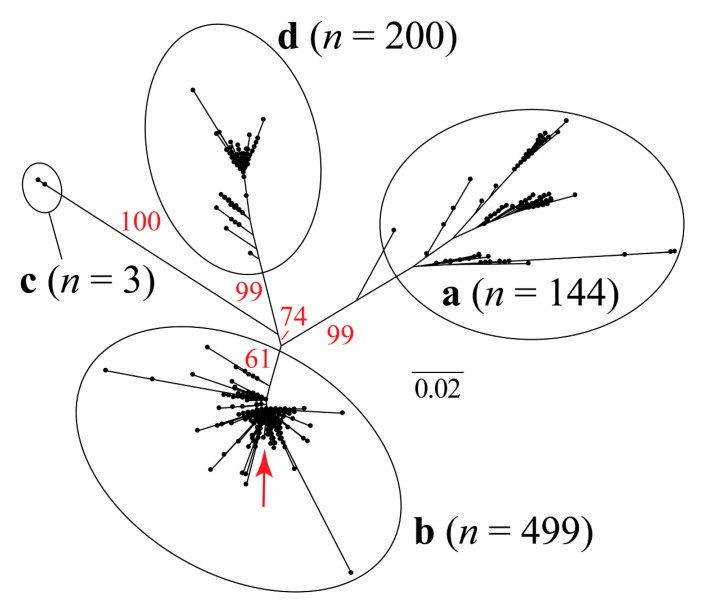
Unrooted maximum likelihood tree based on 705 bp of the porcine circovirus type 2 (PCV2) capsid gene. The red arrow shows the approximate phylogenetic position of the sequence that was recovered in Rancangua in 2007. The genotype classification (a, b, c, and d) follows Franzo et al. (2015) [37] (Supplementary Material, Table S1). In red, the bootstrap values based on 1000 replicates are indicated for the main internal branches.

**Figure 2:**
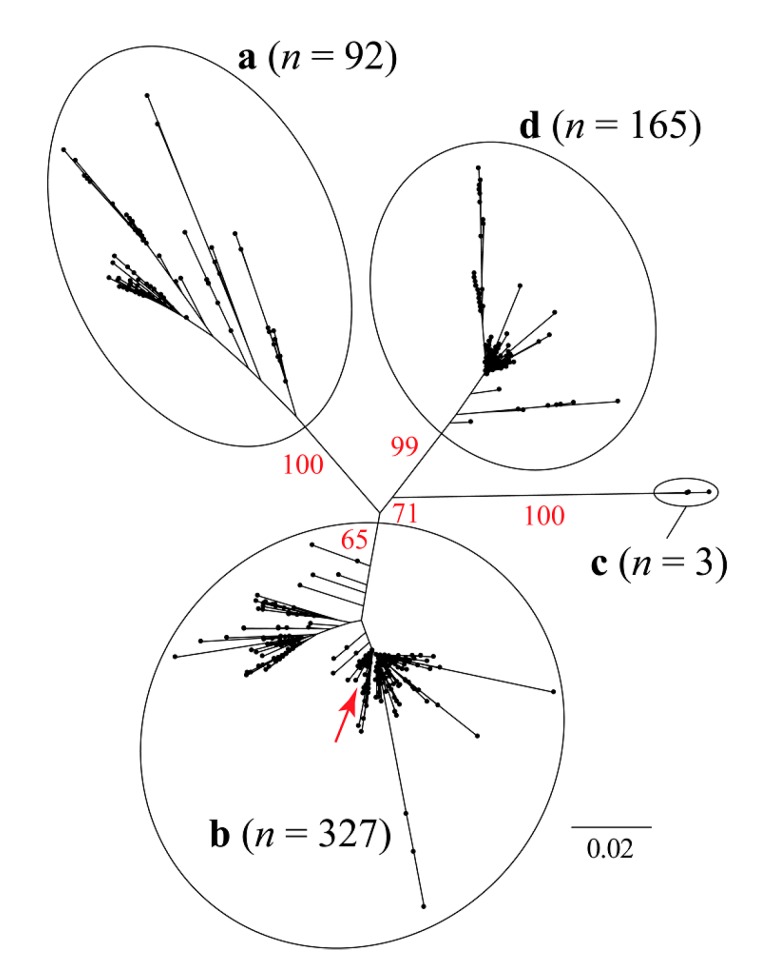
Unrooted maximum likelihood tree based on 1650 bp of the PCV2 capsid + concatenated replicase genes. The red arrow shows approximately the phylogenetic position of the sequence that was recovered in Rancangua in 2007. The genotype classification (a, b, c, and d) follows Franzo et al. (2015) [37] (Supplementary Material, Table S1). In red, the bootstrap values based on 1000 replicates are indicated for the main internal branches.

The maximum likelihood (ML) trees based on a single (capsid) and two concatenated coding genes (capsid and replicase) are shown in Figure 1 and Figure 2, respectively. These analyses resulted in incongruent topologies, that agree with the previously published phylogenetic relationships among different genotypes (a, b, c, and d) of the PCV2 [37,47,48,49].

**Figure 3:**
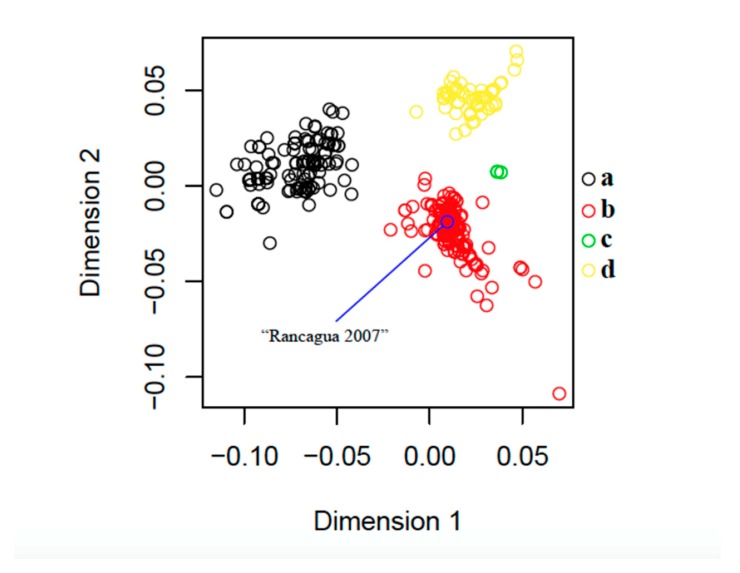
Classical multidimensional scaling (CMDS) based on 846 protein sequences of the PCV2 capsid gene (235 amino acid residues). The blue circle indicates the exact position of the sequence that was recovered in Rancagua in 2007. Following Franzo et al. (2015) [37], genotypes a (*n* = 144), b (*n* = 499), c (*n* = 3), and d (*n* = 200) are shown in black, red, green, and yellow, respectively (Supplementary Material, Table S1).

The sequence isolated in Rancagua in 2007 clusters unequivocally within the PCV2 genotype b clade. Classical multidimensional scaling (CMDS) based on similarity across amino acid residues reveals an equivalent clustering pattern (Figure 3).

Our CMDS analysis exhibits a scattering pattern that clearly separates all four genotypes. The “Rancagua 2007” sample clusters together with a set of 499 additional genotype-b sequences (Figure 3).

### 3.3. Structural Homology for Cap Protein

The structural homolog search for the Cap protein (PDB: 3r0r) was performed using the Topsearch online software. The selected parameters of the structural homolog to be used for the subsequent modeling of the antigenic variant were based on the S, Sq, and St values obtained from the alignment’s analysis (Supplementary Material, Table S2). Three structures were obtained from the analyses accomplished in 2013. From these structures, we selected the capsid protein sequence of the tobacco necrosis virus (PDB: 1c8n), which presented a greater number of similar residues with respect to the PCV2 capsid protein (115) as well as higher similarity and homology values (Supplementary Material, Table S2). Based on this observation, we decided to use these sequences with a length of 276 residues as a homolog in the modeling of the PCV2 Cap protein antigenic variant.

An alignment of the amino acid sequences was performed using the secondary structure of the Cap proteins (PDB: 3r0r) and its structural homolog (PDB: 1c8n) (Supplementary Material, Figure S2). To this end, the information reported for the immunogenic epitopes that included the description of neutralizing antibodies for PCV2 was incorporated [44]. Initially, the N-terminal sequence in grey was removed from the analysis as these sequences are in both PCV1 (avirulent) and PCV2 virus (Supplementary Material, Figure S2). However, these 41 first amino acid residues were added to the Qm1 sequence.

### 3.4. Modeling of the Antigenic Variant Qm1 vs. Cap

Based on the secondary structure alignment that includes the Cap protein and the capsid protein of the tobacco necrosis virus (TNV) (PDB: 1c8n), the regions to be conserved in the structure of the structural homolog chosen from the Cap were analyzed according to the formation of secondary structures, including conserved immunological sequences of the Cap protein (PDB: 3r0r) [24,44].

**Figure 4:**
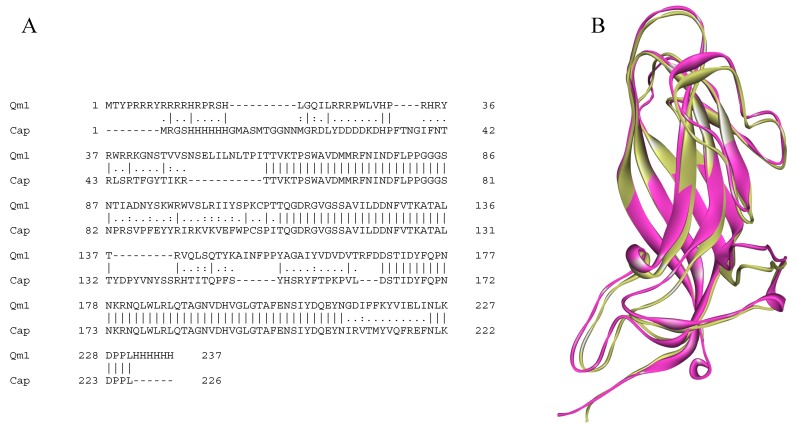
The sequence and structure comparison of the chimera 1 (Qm1) and PCV2 capsid protein (Cap). (**A**) The EMBOSS Needle alignment of the Cap (PDB: 3r0r) and Qm1 protein sequences. The sequences have 59.4% of identity and 67.5% of similarity according to the analysis. (**B**) The structural superposition the crystal structures of PCV2 (pink) and Qm1 (yellow).

**Figure 5:**
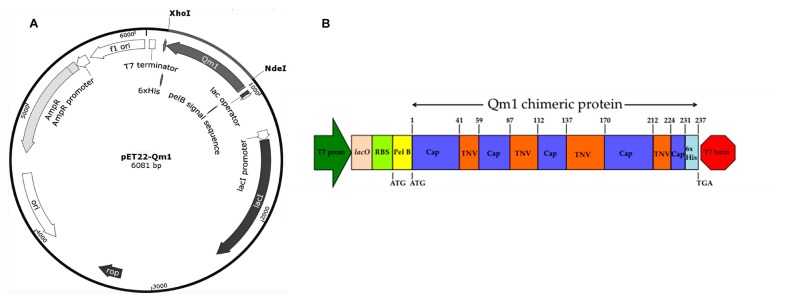
pET22-Qm1 expression plasmid and the genetic engineering design of the chimeric Qm1. (**A**) The Qm1 gene synthesized by GeneScript and inserted between the X*ho*I-N*de*I restriction sites in pET-22b to obtain the pET22-Qm1 final expression vector. (**B**) The genetic engineering design of the chimeric Qm1 protein coded into a bacterial expression cassette. The expression cassette into pET-22b+ shows the T7 promoter in green, and the T7 terminator in red. After the promoter, the genetic elements are the lac operator in pink and the ribosome binding site (RBS) in light green. The pel B leader sequence in yellow is fused to the chimeric protein (Qm1) with the tobacco necrosis virus (TNV) backbone in orange that possess Cap epitopes in blue.

The amino acid sequence of Qm1, represented in yellow, corresponds to the N-terminal conserved region of the 2007 Rancagua isolate (residues: 1–41). The residue sequences 60-87, 112-137, and 168–212 correspond to immunogenic epitopes conserved from the Cap protein (PDB: 3r0r), and the residue sequence 225–231 corresponds to the Cap protein C-terminal followed by a six-histidine tail (residues: 232–237) (Figure 4A and Figure 5B). The remaining Qm1 sequence regions correspond to the structural regions of the TNV capsid protein (PDB: 1c8n) (residues: 42–59, 88–112, 137–170, and 213–224 respectively) (Figure 4A and Figure 5B).

The alignment between both sequences (Qm1 and Cap) showed a similarity of 67.5% and an identity of 59.4% according to the analysis (Figure 4A). Additionally, the superposition of both tertiary structures generated using MODELLER 9.1.1. and visualized by VMD software showed that the designed chimeric protein would adopt the same spatial conformation than PCV2 Cap protein (there was similarity in the backbone of both structures that was supported on the skeleton of the capsid protein of the tobacco mosaic virus in Qm1). Moreover, the Qm1 chimera can maintain immunogenic epitopes from the Cap protein without affecting the spatial arrangement, as well as the N-terminal region from the conserved sequence of the Rancagua2007 isolates shown in yellow (Figure 4B).

### 3.5. pET22b-Qm1 Expression Vector Construction

The codon-optimized Qm1 gene, synthesized by the Genscript company, was inserted in the pET22b (+) vector between the X*ho*I-N*de*I restriction sites (Figure 5A).

The genetic engineering design of the Qm1 protein is detailed in Figure 5B. The Qm1 protein sequence was flanked by the N and C-terminal sequence of the Rancagua 2007 isolates and alternated with the TNV and PCV2 Cap sequences in the intern regions (Figure 5B). The PCV2 Cap sequences in the middle of the Qm1 design correspond to the most immunogenic epitopes of the Cap. The Qm1 sequences were inserted just behind the pelB region of the pET22b (+), and at the end of the sequences a His-tag was added (Figure 5B).

### 3.6. Qm1 Expression in SHuffle^®^ T7

**Figure 6:**
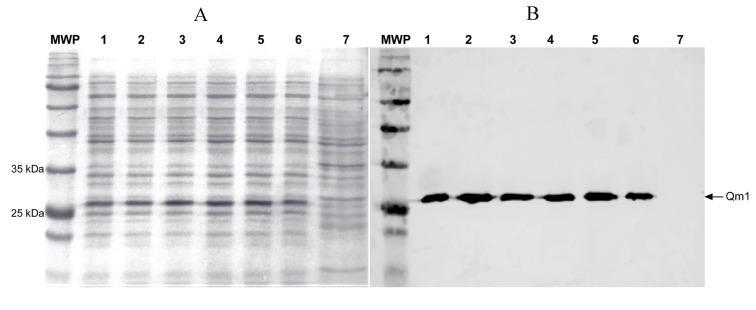
The expression analysis of Chimera 1 (Qm1) in *Escherichia coli* (SHuffle^®^ T7 Express) under denaturant conditions. SDS-PAGE (**A**) and Western blot assay (**B**) using monoclonal anti-histidine antibodies (Clontech). MWP: molecular weight pattern, 1–6: six transformant colonies. 7: untransformed SHuffle^®^ T7 Express *E. coli* strain used as a negative control. All samples were processed after 6 h of induction.

The SHuffle^®^ T7 Express *E. coli* strain was transformed with the pET22-Qm1 expression plasmid. The clone selection was performed on a LB plate with ampicillin. Six transformant colonies were grown in liquid LB media and their expression was induced with IPTG. Qm1 was evaluated by SDS-PAGE and Western blotting at 6 h post-induction. The SDS-PAGE results showed a reinforced band around 27 kDa in all colonies (lanes 1–6), except in the negative control (lane 7) (Figure 6).

The identity of the Qm1 protein in this band was confirmed by Western blot analysis using an anti-His-tag antibody from Takara Bio (Mountain View, CA, USA) (Clontech) (Figure 6), where the presence of the Qm1 protein was observed in all the colonies (lanes 1–6) while no signal was detected in the negative control (lane 7). This result confirms the correct induction and expression of the His-tagged Qm1 protein.

### 3.7. Qm1 Specific Antibody Detection

**Figure 7:**
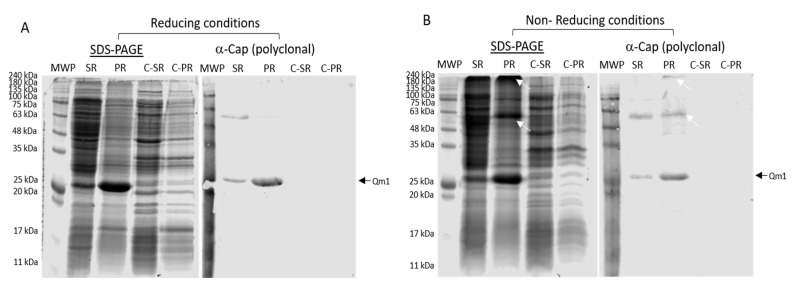
The identification of Qm1 expressed in the SHuffle^®^ T7 Express *E. coli* strain. (**A**) SDS-PAGE Coomassie blue staining in the reducing condition (left) and Western blot (right) using a polyclonal anti-Cap antibody, previously released against proteins of the SHuffle^®^ T7 Express strain of *E. coli*. (**B**) The same as (**A**), but SDS-PAGE under non-reducing conditions. MWP: molecular weight pattern; SR: supernatants of cell rupture; PR: pellet of cell rupture of the Qm1 clone 1. C-SR and C-PR correspond to the negative control sample of the SHuffle^®^ T7 Express. The white arrows indicate protein aggregates detected by the antibody. A volume of 20 µL per sample was used in each case in both conditions.

To analyze the correct recognition of the Qm1 protein using a specific commercial antibody against the Cap protein of PCV2, we used clone 1 obtained from the previous expression analysis (Figure 7).

The SDS-PAGE analysis of the supernatant and the pellet after the cell disruption of the cells of clone 1 under reducing conditions showed an increased signal at approximately 27 kDa, both in the supernatant and pellet, observed with greater intensity in the rupture pellet with respect to the negative control samples (Figure 7A SDS-PAGE). The Western blot analysis under these conditions exhibited recognition by a commercial anti-Cap polyclonal antibody at the expected molecular weight of the band, according to the design of the Qm1 (≈27 kDa) (Figure 7A α-Cap (polyclonal)).

**Figure 8:**
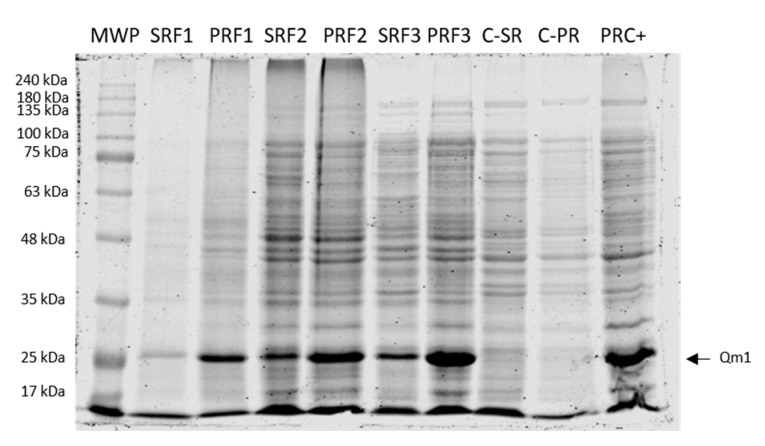
SDS-PAGE of the Qm1 expression in fermentation. SDS-PAGE with Coomassie blue staining under reducing conditions after 6 h of induction. MWP: molecular weight pattern; SRF1, SRF2, and SRF3: rupture supernatants from each of the three fermentations of the Qm1 with SHuffle^®^ T7 Express clone; PRF1, PRF2, and PRF3: the pellet of rupture from each of the three fermentations of Qm1 SHuffle^®^ T7 Express clone; C-SR and C-PR: the rupture supernatants and pellet of rupture from the fermentation negative control sample. PRC +: the previous sample obtained positive detected by Western blot with an anti-his antibody. We used 20 µL per sample.

The same experiment, performed under non-reducing conditions (Figure 7B), showed similar results to those obtained under reducing conditions, where a band was observed in SDS-PAGE, corresponding to 27 kDa, in addition to a signal corresponding to the dimer of Qm1 (≈55 kDa), plus a signal at the top of the gel corresponding to a high molecular weight (>240 kDa) unlike that observed under reducing conditions (Figure 8). The Western blot assay under non-reducing conditions corroborated the correct recognition of the 27, 55, and more than 240 kDa (high molecular weight) bands, with the highest molecular weights observed in the rupture pellet of the cells from clone 1 (Figure 7, white arrows).

### 3.8. Qm1 Batch Fermentation

To assess chimeric protein Qm1 expression in the controlled condition, the clone 1 culture was performed in a 5 L Winpact bioreactor with triplicate consecutive batches performed. After induction for 6 h, the Qm1 expression was analyzed in the supernatant and the pellet of disrupted cell fractions in each case (Figure 8).

The SDS-PAGE results showed an intense signal at approximately 27 kDa (as expected), with the highest band corresponding to the rupture pellet of F3 (Figure 8, PRF3). In addition, the signal disappears in the fractions analyzed for a negative control fermentation (SHuffle^®^ T7 Express *E. coli* strain without transformation) (Figure 8 C-SR and C-PR).

The performance parameters corresponding to fermentations F1, F2, and F3 are summarized in Table 1. According to these results, the biomass average of the three fermentations resulted in 8.4 ± 1.7 g of dry weight per 5 L after 6 h of induced culture and a total culture time of 8 h of culture.

The Qm1 percentage of the total protein in the insoluble fraction was 8.7% ± 1.1% and the volumetric productivity was 2.8 ± 0.8 mg/L/h.

### 3.9. Qm1 Protects Pigs against PCV2 Infection

The immunogenicity of Qm1 and its protection capacity against PCV2 infections was evaluated in pigs. Initially, we evaluated the formulation of reactogenicity in pigs with single or triple doses in groups of 10 animals each. The time-course of rectal temperature values showed no significant differences between groups (Supplementary Material, Figure S4). The inoculation area did not show reactivity or irritation in any animal during the experiment, and we observed normal appetite and behavior in all groups.

**Figure 9:**
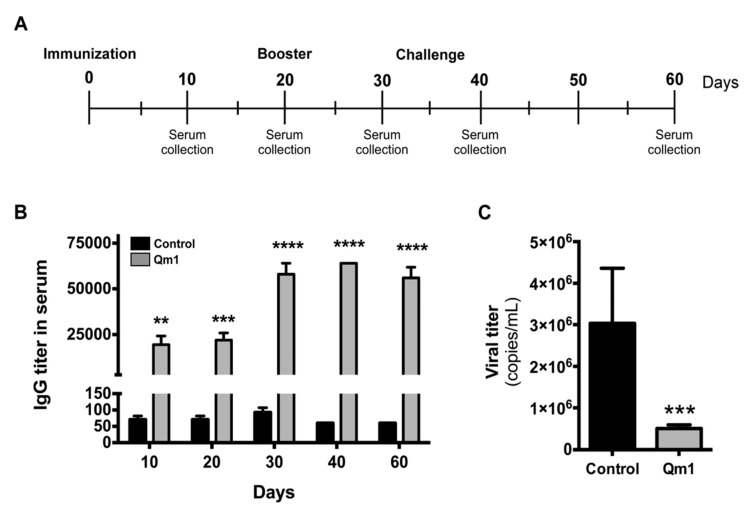
Analysis of the antibody and antiviral response to PCV2 in pigs. (**A**) The immunization and viral challenge schedule. (**B**) IgG titer in serum measured in pigs vaccinated with Qm1 (grey) or the vehicle PBS (black) by indirect ELISA at the indicated days. (**C**) The pig serum was collected at day 60 and the viral titer was quantified in challenged pigs by qRT-PCR using SensiMixTM SYBR Hi-ROX kit (Bioline). The values are the mean ± standard error mean (SEM). n = 6, two-way ANOVA and the Sidak test for IgG titer and the Student’s *t*-test for viral titer ** *p* < 0.01; *** *p* < 0.001; **** *p* < 0.0001 vs. the control group.

The pigs were immunized with 100 μg of Qm1 using the intramuscular route, while saline solution (0.9% (m/v) NaCl) was used as a negative control. The immunization assay and the challenge with the virus were executed as described in Figure 9A.

The anti-Cap IgG titer was measured by indirect ELISA, and it was significantly higher in the serum of pigs immunized with Qm1, compared to the control group, starting at day 10 and reaching a maximum of 1:64,000 (Figure 9B).

The pigs were challenged with PCV2 at day 35 and the viral titer was measured at day 60 by qPCR (copies viral DNA/mL serum). These results indicate that Qm1 protects pigs against the virus, reducing the viral titer in the serum to 5 × 10^5^ copies/mL, compared with the control group at 3 × 10^6^ copies/mL (Figure 9C).

## 4. Discussion

### 4.1. Design of Qm1 Chimeric Antigen

The design of the chimeric protein was based on the assembly of a molecule with structural and immunogenic characteristics similar to that of the capsid protein of PCV2, for its use as the active principle in an efficacious vaccine against this virus. The sequence used to belong to a virulent strain isolated from a natural outbreak and corresponds to a Chilean PCV2b genotype (Figure 1, Figure 2 and Figure 3), the most epidemic porcine circovirus described in other countries [50]. The newly designed protein has a conserved N-terminal region common to several Chilean isolates, as well as other variants of the same isolated virus. This characteristic could expand the use of the vaccine in other areas of the world where different isolates of the PCV2 prevail. The inclusion of immunogenic sequences described for the Cap protein of PCV2 will guarantee the generation of an immune humoral response against the native Cap protein, which will contribute to the elimination of the virus in animals [44].

The use of regions of a protein homologous to Cap from PCV2 as a backbone (the capsid protein of TNV, PDB: 1c8n), allowed for the design of a structurally similar protein to that of the PCV2 capsid, however, with a much lower sequence identity, about 66% regarding the PCV2 Cap, according to sequence alignment (Figure 4A). The overlapping of the 3D-structures of the proteins (Qm1 and Cap), showed a high spatial structure similarity (Figure 4B). In this way, the generation of a protein with a similar folding to the PCV2 Cap protein using bioinformatics tools allows the use of this chimeric protein as a potential antigen in the generation of vaccines against PCV2, containing the most exposed antigenic domains [44]. The inclusion of a His-tag allows its immune-identification and further purification using immobilized metal affinity chromatography (IMAC) (Supplementary Material, Figure S3).

The new chimeric protein, Qm1, is the first reported plant virus capsid based on a molecule with these characteristics assembled for its use as an active principle for a subunit veterinary vaccine. Currently marketed vaccines, used to prevent the infection of herds with PCV2, are based on the capsid protein expressed in insect cell cultures and/or attenuated viruses [51,52]. Moreover, there are no current reports on the use of a similar design to the one described in our work for the generation of a protein for this purpose. There are currently at least four commercial PCV2 vaccines, two based on the Cap protein from insect cells culture (Circumvent^®^ (MSD Animal Health) and Ingelvac CircoFLEX^®^ (Boehringer Ingelheim) and two are based on an inactivated variant of the virus: Circovac^®^ (Merial) and Fostera^®^ (Zoetis) [53]. The Baculovirus-based expression system has several advantages, including the correct folding of different proteins, the assembly of correct glycosylation patterns, and the co-expression of several proteins. However, the main drawback of this system is the high cost associated with the manufacturing of the recombinant protein, which is significantly more expensive than the bacterial or yeast-based expression systems [54].

### 4.2. Anti-Cap Antibody Recognition

One of the uncertainties of the chimeric protein original design was whether it would be recognized by antibodies against the native Cap antigen after expression in a simple and inexpensive system like *E. coli*. This work showed initially the new molecule recognition using an anti-His tag antibody (Figure 6), which allowed us to follow its presence along with the further expression and purification steps. The expression of the Qm1 was confirmed using a commercial polyclonal anti-Cap antibody by the presence of a positive band at the expected molecular weight of ≈27 kDa in both the supernatant and the pellet of disrupted cells (Figure 7). This result was obtained under reduced and non-reduced SDS-PAGE (Figure 7A,B), which is in correspondence with previous reports for the Cap protein expressed in *E. coli* [27,55]. The recognition of much higher molecular weight bands (more significant in the case of non-reducing conditions corresponding to the dimer (54 kDa) and multimers (>240 kDa) (Figure 7)) confirms that the chimeric protein Qm1 forms high-molecular-weight aggregates, such as the PCV2 capsid, reported for the native Cap protein expressed both in *E. coli* and yeast [26,27,56].

Along with this work, the Qm1 largest fraction protein was found in the insoluble fraction as inclusion bodies (Figure 7). This is presumably due to the overexpression of the heterologous gene, leading to the formation of intracellular inclusion bodies, which can make up to 95% of the recombinant protein [57] and can be easily separated by centrifugation due to their higher density [58]. These protein aggregates confer a defense mechanism to the proteins against proteolytic degradation [59]. In some cases, the expressed proteins are cytotoxic and affect the cell growth, thus protein aggregates as inclusion bodies lower this toxicity, alleviating the issue of culture viability [60]. The advantages of inclusion bodies, such as high purity, easy purification, and resistance against proteolysis, has made the potential use as candidates for vaccine formulation an interesting option [61].

### 4.3. Qm1 Production

The average biomass obtained for the SHuffle^®^ T7 Express *E. coli* clone, which expressed Qm1 from three consecutive fermentations, was 1.7 g/L of dry weight (Table 1, 8.4 g per 5 L). This value is in the order of magnitude for similar reports for *E. coli* cultures, with a final OD600 = 2.0 at the end of the fermentation [62]. The Qm1 protein concentration was approximately 22 mg/L, which represents 8.7% ± 1.1% of the total proteins after cell disruption, when analyzing only the non-soluble fraction. This is, to the best of our knowledge, the only expression report for the SHuffle^®^ T7 Express *E. coli* strain in the scientific literature. The production results of this work are below those reported for the classical *E. coli* expression systems (e.g., BL21 Codon Plus), with 10%–50% of the total proteins [63,64]. However, considering both fractions in the analysis, as well as the fact that Qm1 forms high-molecular-weight aggregates (Figure 7), the yield would be similar to what was previously reported for *E. coli*.

The SHuffle^®^ T7 Express strain was chosen in order to obtain the protein mostly in the soluble fraction. However, along the expression process and under the culture conditions and cell disruption conditions tested, as well as the disruption methodology used, the Qm1 main fraction was identified in inclusion bodies (Figure 7 and Figure 8), which is of major interest for its use as a vaccine candidate. The protein of interest was obtained in both fractions using the SHuffle^®^ T7 Express strain, which has been described by different authors, where its distribution in both soluble and insoluble fractions depends on the characteristics and complexity of the protein, as well as the combinations of culture parameters, such as the composition of the culture medium, inducer concentration, culture time, and temperature, among others [65,66,67,68].

In this work, the protein was obtained mostly in the insoluble fraction, using typical growth conditions for *E. coli*, with a temperature of 37 °C and 6 h of induction time, which simplifies the process and allowed us to obtain higher yields than the typical SHuffle^®^ T7 Express protocols at 30 °C, where a longer culture time is required to achieve the same results (longer induction times of 12–24 h) [69]. The Qm1 expression level observed was obtained from fermentation in triplicate (F1, F2, and F3) (Table 1).

### 4.4. Protection against PCV2 Induced by the Qm1 Antigen

The ELISA-determined immunoglobulin G (IgG) titer for PCV2 in the sera of vaccinated piglets was in the order of 1:64,000 on day 30 after vaccination, and this value was constant until day 60. These Qm1-related IgG sera titers for the vaccinated piglets were higher than those reported for subcutaneously vaccinated mice with a PCV2-encoding VLP-based vaccine, with a 1:40,000 titer, as well as with the CircoFLEX^®^ vaccine, where the titer was below 1:10,000 [26]. A DNA vaccine, expressing the Cap protein for PCV2, also showed an increase in IgG in the sera of vaccinated piglets, however, the titer was not calculated and it is therefore not comparable with the results of this work [70].

After challenge, there were no significant variations in the titers of anti-PCV2 antibodies in animals, neither in the vaccinated group nor in the control group. This finding is associated with the fact that PCV2 generates a suppression of the immune system of non-vaccinated, infected animals [26,71,72]. The viral titers corresponding to piglets vaccinated with Qm1 were significantly lower at the end of the assay regarding the control group; however, the viral titer is still in the order of 10^6^ (Figure 9).

Our results indicate that the chimeric variant Qm1 is a potential vaccine candidate against the PCV2 virus infection. However, additional trials with a larger number of animals, with a longer duration, and also comparing these results with those of a commercial anti-PCV2 vaccine are necessary. Similar results were obtained using mice as an animal model, where two types of vaccines were compared: PCV2-derived VLPs and Ingelvac-CircoFLEX^®^ [26]. However, in a study where the anti-PCV2 antibody titer in pigs immunized with Baculovirus-expressed Cap was compared to the commercial vaccine Ingelvac-CircoFLEX^®^, the anti-PCV2 antibody ELISA-titers were lower than those of this work (Supplementary Material, Figure S5), as well as for forthcoming days considering absorbance at 492 nm (day 21) [73].

Conversely, a report comparing the antibody response of piglets of two commercial vaccines against PCV2 SuiShot^®^ Circo-ONE and Ingelvac-CircoFLEX^®^ showed that when quantifying the anti-PCV2 antibodies, a significant difference regarding the control at day 50 after vaccination was detected [74]. The results obtained in this work demonstrated increased production of the total antibodies at day 10 after vaccination, which could suggest an earlier response and protection against the viral infection when compared to other vaccines. On the other hand, most of the currently marketed veterinary vaccines against PCV2 are based on the use of an insect cell-based expression system to obtain the Cap protein as an active principle, such as Circumvent^®^ (MSD Animal Health-NAFTA) and Ingelvac CircoFLEX^®^ (Boehringer Ingelheim), while other vaccines are based on the use of an attenuated virus, e.g., Circovac^®^ (Ceva) and Fostera^®^ (Zoetis), from infected cells or animal embryos [75,76].

Inactivated vaccines usually do not confer as strong of protection as other vaccines. Therefore, it is possible that several doses of the vaccine or booster will be necessary to provide continuous immunity against the diseases. These methods share a long and expensive biotechnological process, due to the use of complex eukaryotic cells, leading to the acquisition of the necessary complex equipment and infrastructure to develop this product. Conversely, the processes associated with recombinant vaccines in *E. coli* are easier to develop, simpler to operate, and generally cheaper than those of the more complex expression systems. There are several examples of recombinant *E. coli*-based vaccines [77,78,79].

Currently, *E. coli* is the most frequently used microorganism for recombinant protein production, due to its easy manipulation, standardized and optimized culture techniques, fast growth, and low maintenance cost, with a great variety of biopharmaceutical products expressed and USDA-approved in this system [80,81]. On the other hand, Baculovirus cell cultures, even if they are able to generate proteins with complex post-translational modifications, are inherently expensive due to the media necessary to support their growth along with the infection phase, and therefore the expression of the protein of interest [54].

For an effective PCV2 vaccine, as shown in this study, the Qm1 protein does not require the post-translational modifications generated by the more complex eukaryotic cells, therefore the recombinant *E. coli* system seems to be an optimal microorganism for the synthesis of this protein. This allows a high expression level and low cost of goods. Furthermore, most of the marketed PCV2 vaccines are based on VLPs, whose purification process is time-demanding and expensive [54,82], while on the contrary Qm1, which is expressed as an inclusion body, minimizes the purification steps that could increase the process-associated costs.

## 5. Conclusions

The implemented design allowed us to generate a biologically active molecule that was efficiently expressed in the *E. coli* system for use as a vaccine candidate. Our molecular design proposed in this work could be used to express the domains or fragments of other antigenic proteins, whose structural complexity does not allow for low-cost manufacturing in *E. coli*. Many other antigenic protein domains could be integrated into the TNV backbone, reaching the necessary immunogenicity and antigenic identity, similar to those obtained in this project. A potential example of this is the E2 antigen of the classical swine fever virus (CSFV), whose recombinant expression is currently restricted to mammalian cells to ensure its antigenicity [83].

## Figures and Tables

**Table 1:** The performance parameters of three fermentations of Qm1 expression in SHuffle^®^ T7 Express *E. coli* strain after 8 h of culture.

Fermentation	Total Biomass (g in 5 L)	Total Protein (mg/g Of Disrupted Biomass)	Qm1 (mg/g Of Disrupted Biomass)	% Qm1 (Of Protein)	Qm1 Total (mg)	Qm1 Productivity (mg/L/h)
F1	10.6	32.0	2.83	8.8	107.6	2.7
F2	6.4	42.7	3.10	7.3	71.3	1.8
F3	8.1	52.9	5.24	9.9	152.0	3.8
Average	8.4 ± 1.7	42.5 ± 8.5	3.7 ± 1.1	8.7 ± 1.1	110.3 ± 33	2.8 ± 0.8

## Supplementary Materials

The following are available online at https://www.mdpi.com/1999-4915/12/4/385/s1, Figure S1: Sequence of a viral isolate of a representative Chilean strain. Figure S2: Sequence alignment of secondary structure information. Figure S3: Qm1 purification by IMAC. Figure S4: Rectal temperature assay. Figure S5: Response to Qm1 vaccination in pigs. Table S1: Sequences of porcine circovirus type 2 (PCV2) included in this study. Table S2: Structural analysis by online TopSearch software.
