# Supplementary material for: A Heterologous Viral Protein Scaffold for Chimeric Antigen Design: An Example PCV2 Virus Vaccine Candidate
Source: Viruses. 2020 Mar 31;12(4):385. doi: 10.3390/v12040385 (PMC7232224; doi:10.3390/v12040385)
Supplement: Supplementary file 1 [file viruses-12-00385-s001.pdf]

Supplementary materials.

>PCV2 Chile Rancagua2007 Cap gene
ATGACGTATCCAAGGAGGCGTTACCGGAGAAGAAGACACCGCCCCCGCAGCCA
TCTTGGCCAGATCCTCCGCCGCCGCCCCTGGCTCGTCCACCCCCGCCACCGTTA
CCGCTGGAGAAGGAAAAATGGCATCTTCAACACCCGCCTCTCCCGCACCTTCG
GATATACTATCAAGCGAACCACAGTCAAAACGCCCTCCTGGGCGGTGGACATG
ATGAGATTCAATATTAATGACTTTCTTCCCCCAGGAGGGGGCTCAAACCCCCGC
TCTGTGCCCTTTGAATACTACAGAATAAGAAAGGTTAAGGTTGAATTCTGGCCC
TGCTCCCCGATCACCCAGGGTGACAGGGGAGTGGGCTCCAGTGCTGTTATTCTA
GATGATAACTTTGTAACAAAGGCCACAGCCCTCACCTATGACCCCTATGTAAAC
TACTCCTCCCGCCATACCATAACCCAGCCCTTCTCCTACCACTCCCGCTACTTTA
CCCCCAAACCTGTCCTAGATTCCACTATTGATTACTTCCAACCAAACAACAAAA
GAAATCAGCTGTGGCTGAGACTACAAACTGCTGGAAATGTAGACCACGTAGGC
CTCGGCACTGCGTTCGAAAACAGTATATACGACCAGGAATACAATATCCGTGT
AACCATGTATGTACAATTCAGAGAATTTAATCTTAAAGACCCCCCACTTAACCC
TTAA

>PCV2 Chile Rancagua2007 Cap protein
MTYPRRRYRRRRHRPRSHLGQILRRRPWLVHPRHRYRWRRKNGIFNTRLSRTFGY
TIKRTTVKTPSWAVDMMRFNINDFLPPGGGSNPRSVPFEYYRIRKVKVEFWPCSPIT
QGDRGVGSSAVILDDNFVTKATALTYDPYVNYSSRHTITQPFSYHSRYFTPKPVLD
STIDYFQPNNKRNQLWLRLQTAGNVDHVGLGTAFENSIYDQEYNIRVTMYVQFRE
FNLKDPPLNP*

Figure S1. Sequence of a viral isolate of a representative Chilean strain. Up: nucleotide sequence of the gene, "PCV2 Chile Rancagua2007 Cap gene “. Down: amino acid sequence of the Cap protein, "PCV2 Chile Rancagua2007 Cap protein"

Table S1. Sequences of porcine circovirus type 2 (PCV2) included in this study. Genbank accession numbers (GAN) and genotypes (G) according to Franzo *et al*. (2015). Asterisk (*) indicates that both capsid and replicase gene sequences were obtained from the same Genbank entry and used in the concatenated data set.

GAN	G	GAN	G	GAN	G	GAN	G	GAN	G	GAN	G
AB361566	b	AY122275*	b	AY484412*	b	DQ218419*	b	EF190943	b	EF524529*	b
AB361567	b	AY129154	a	AY484413*	b	DQ218420*	b	EF197987	b	EF524539*	d
AB361568	a	AY129155	a	AY484414*	b	DQ218421*	b	EF210106*	b	EF560608	b
AB361569	b	AY146991*	a	AY484415*	b	DQ220733*	b	EF371518	a	EF560609	b
AB361570	a	AY146992*	a	AY484416*	b	DQ220737*	b	EF371519	a	EF560610	b
AB361571	a	AY146993*	a	AY510375*	d	DQ220738*	b	EF371520	a	EF565342*	b
AB361572	a	AY180396*	a	AY536755*	b	DQ233257*	b	EF371521	a	EF565344*	b
AB361573	a	AY180397*	a	AY536756*	b	DQ364650*	b	EF371522	a	EF565346*	b
AB361574	a	AY181945*	b	AY556473*	d	DQ397521*	a	EF371523	a	EF565347*	b
AB361576	a	AY181947*	d	AY556475*	b	DQ534442	b	EF371524	a	EF565349*	b
AB361577	a	AY181948*	a	AY556476*	d	DQ629113*	a	EF371525	a	EF565350*	b
AB361582	a	AY188355*	b	AY578327	b	DQ629114*	a	EF371528	b	EF565352*	b
AB361583	a	AY217743*	b	AY579893*	b	DQ648031*	b	EF371529	b	EF565354*	b
AB361584	b	AY256455*	a	AY596822	b	DQ861898*	b	EF371530	b	EF565355*	b
AB361585	b	AY256456*	a	AY596823*	b	DQ861900*	b	EF371531	b	EF565356*	b
AB426905*	a	AY256457*	b	AY604430*	b	DQ861901*	b	EF371532	b	EF565357*	b
AB462382	b	AY256458*	a	AY613854*	b	DQ870484*	a	EF371533	b	EF565358*	b
AB462383	a	AY256459*	a	AY613906	b	DQ910865*	b	EF371534	b	EF565359*	b
AB462384	d	AY256460*	b	AY641542*	b	DQ910866*	b	EF371535	b	EF565360*	b
AB462385	a	AY288133*	b	AY651850*	b	DQ915584*	b	EF371536	b	EF565361*	b
AB462387	b	AY288134*	b	AY672600	a	DQ915585*	b	EF371537	b	EF565362*	b
AB462388	a	AY288135*	a	AY672601	a	DQ915586*	b	EF371538	b	EF565363*	b
AB462389	b	AY291316*	b	AY678532*	b	DQ915587*	b	EF371539	b	EF565364*	b
AB462390	b	AY291317*	d	AY682990*	b	DQ915588*	a	EF371540	b	EF565366*	b
AB462391	b	AY321982*	b	AY682991*	d	DQ997815*	b	EF371541	b	EF565367*	b
AF201305*	a	AY321983*	b	AY682992*	b	EF064149	b	EF371542	b	EF565368*	b
AF201306*	a	AY321984*	b	AY682994*	d	EF064150	a	EF371544	b	EF592575*	b
AF201307*	a	AY321985*	b	AY682996*	d	EF067852	b	EF371546	b	EF592576*	b
AF201308*	a	AY321986*	b	AY686762*	b	EF067853	a	EF371548	b	EF619037*	b
AF201309*	a	AY321987*	b	AY686763*	d	EF184220	a	EF371550	b	EF619971	b
AF201310*	a	AY321988*	b	AY686764*	b	EF184221	a	EF371551	a	EF675230*	d
AF201311*	b	AY321989*	b	AY686765*	d	EF184222	a	EF394774*	a	EF675234*	b
AF264038*	a	AY321990*	b	AY691169*	b	EF184223	a	EF394775*	a	EF675235*	b
AF264039*	a	AY321991*	b	AY691679*	b	EF184224	a	EF394776*	a	EF675236*	b
AF264040*	a	AY321992*	b	AY699793*	a	EF184225	a	EF421967*	b	EF675237*	b
AF264041*	a	AY321995*	b	AY732494*	b	EF184226	b	EF421968*	b	EF675239*	b
AF264042*	a	AY321996*	b	AY754022*	a	EF184227	b	EF421969*	b	EF675241*	d
AF264043*	a	AY321997*	b	AY849938*	b	EF184228	b	EF421970*	b	EF675242*	b
AF381175*	a	AY321999*	b	AY874163*	b	EF190922	b	EF421971*	b	EF675244*	b
AF381176*	a	AY322000*	b	AY874164*	a	EF190923	b	EF421973*	b	EF990645	d
AF381177*	a	AY322002*	b	AY874165*	a	EF190924	b	EF452350*	b	EU136711*	a
AF454546*	a	AY322003*	b	AY874166*	a	EF190925	b	EF467928	b	EU136712*	b
AF465211*	a	AY322004*	a	AY874168*	b	EF190926	b	EF493837*	b	EU136713*	b
AF520783*	a	AY325495*	a	AY885225	b	EF190928	b	EF493838*	b	EU136714*	b
AF544024*	a	AY424401*	a	AY916791*	b	EF190929	b	EF493839*	b	EU136715*	b
AJ623306*	b	AY424402*	a	AY943819*	d	EF190930	b	EF493840*	b	EU136716*	b
AY094619*	a	AY424403*	a	DQ104419	a	EF190932	b	EF493841*	b	EU136717*	b
AY099495*	a	AY424404*	b	DQ104421*	a	EF190933	b	EF493842*	b	EU136718*	b
AY099496*	a	AY424405*	b	DQ104422*	b	EF190934	b	EF524515*	b	EU136719*	b
AY099497*	a	AY484407*	b	DQ104423*	a	EF190935	b	EF524519*	b	EU136720*	b

AY099498*	a	AY484408*	b	DQ195679*	b	EF190937	b	EF524521*	b	EU148503*	c
AY099499*	a	AY484410*	d	DQ201641*	b	EF190939	b	EF524525*	b	EU148504*	c
AY099500*	a	AY484411*	b	DQ201642*	b	EF190942	b	EF524527*	b	EU148505*	c

GAN	G	GAN	G	GAN	G	GAN	G	GAN	G	GAN	G
EU148507*	a	EU886637*	b	FJ667587*	b	GU247987*	b	HM142894*	d	HQ395029*	d
EU257512*	b	EU909686*	d	FJ667588*	d	GU247988*	b	HM142895*	d	HQ395032*	d
EU257513*	b	EU909688*	b	FJ667589*	b	GU247989*	b	HM142896*	d	HQ395033*	d
EU257514*	b	EU980089	b	FJ667590*	d	GU247990*	b	HM142899*	d	HQ395036*	b
EU257515*	b	EU980090	b	FJ667591*	b	GU247991*	b	HM161710*	d	HQ395037*	d
EU283329*	b	EU980094	b	FJ667592*	b	GU247992*	b	HM161711*	d	HQ395038*	d
EU296794	b	FJ158603*	b	FJ667593*	b	GU252370*	d	HM535639	b	HQ395039*	b
EU346945*	b	FJ158604*	d	FJ667594*	b	GU325760*	d	HM535640	d	HQ395041*	d
EU350548	b	FJ158606*	b	FJ667595*	b	GU325762*	d	HM535641	d	HQ395042*	d
EU366324*	b	FJ158607*	d	FJ667596*	b	GU325763*	d	HM565923	b	HQ395043*	d
EU366325*	b	FJ218001*	a	FJ712215*	d	GU325764*	b	HM565924	b	HQ395044*	d
EU366326*	b	FJ233908*	a	FJ712216*	d	GU325766*	b	HM623764*	a	HQ395046*	b
EU408780*	b	FJ384965	b	FJ716703*	b	GU325767*	d	HM641752*	b	HQ395048*	d
EU418627*	b	FJ384966	b	FJ716704*	b	GU325768*	d	HM755880	d	HQ395050*	d
EU503031*	b	FJ384967	b	FJ755686	b	GU325769*	d	HM755881	d	HQ395052*	d
EU503032*	b	FJ384968	b	FJ870967*	a	GU325770*	d	HM776437*	d	HQ395053*	d
EU503033*	b	FJ384969	b	FJ870968*	a	GU370064*	a	HM776438*	b	HQ395054*	a
EU503034*	b	FJ426398*	d	FJ870969*	b	GU574204	d	HM776439*	d	HQ395055*	d
EU503035*	b	FJ483938*	a	FJ870970*	d	GU808525*	d	HM776440*	d	HQ395057*	b
EU503037*	b	FJ501957*	a	FJ870971*	d	GU938302*	d	HM776441*	d	HQ395059*	d
EU503038*	b	FJ594471*	d	FJ870972*	d	GU938303*	b	HM776442*	b	HQ395061*	d
EU503039*	b	FJ598044*	b	FJ870973*	d	HF542107*	b	HM776444*	b	HQ591365*	b
EU503040*	b	FJ598045*	d	FJ870974*	b	HM003569*	b	HM776445*	b	HQ591366*	a
EU518247	b	FJ623185*	b	FJ870976*	b	HM003570*	b	HM776446*	b	HQ591367*	b
EU521707*	b	FJ644555	b	FJ905462*	b	HM009329	b	HM776447*	d	HQ591368*	b
EU521708*	b	FJ644556	b	FJ905464*	b	HM009332	b	HM776448*	b	HQ591370*	b
EU521709	b	FJ644557	b	FJ905468*	b	HM009333	b	HM776449*	d	HQ591371*	b
EU545543*	b	FJ644558	b	FJ905469*	b	HM009334	b	HM776451*	b	HQ591372*	b
EU545544*	b	FJ644559	b	FJ905471*	a	HM009335	b	HM776453*	d	HQ591373*	b
EU545545*	b	FJ644560	d	FJ935780*	b	HM009336*	b	HQ113117*	d	HQ591374*	a
EU545546*	b	FJ644561	b	FJ948167*	b	HM009337*	a	HQ113118*	d	HQ591375*	b
EU545547*	b	FJ644562	b	FJ948168*	d	HM027579*	d	HQ113119*	d	HQ591376*	b
EU545548*	b	FJ644563	b	FN398023*	b	HM027580*	d	HQ113120*	b	HQ591377*	b
EU545549*	b	FJ644919	b	FN398025*	b	HM030908	b	HQ113121*	b	HQ591378*	b
EU545550*	b	FJ644920	b	FN398026	b	HM038016*	b	HQ231328*	b	HQ591380*	b
EU545551*	b	FJ644921	b	FR823451*	a	HM038017*	d	HQ378157	d	HQ650833*	d
EU555439*	b	FJ644922	b	GQ174519*	b	HM038018*	b	HQ378158*	d	HQ693092	b
EU589623	b	FJ644923	b	GQ227412*	b	HM038019*	b	HQ378159*	b	HQ693093*	d
EU656143*	b	FJ644924	b	GQ359008*	b	HM038020*	b	HQ378161*	b	HQ735207	b
EU684164*	b	FJ644925	b	GQ359009*	d	HM038021*	b	HQ378162	b	HQ738639*	b
EU747125	a	FJ644926	b	GQ359010*	d	HM038022*	b	HQ378163	b	HQ738640*	b
EU755371	b	FJ644929	d	GQ359011*	d	HM038023*	b	HQ378164	a	HQ738641*	b
EU755372	b	FJ644930	b	GQ449672*	b	HM038024*	b	HQ378165	a	HQ831519*	a
EU755373	b	FJ644931	d	GQ915288*	d	HM038025*	b	HQ378166	a	HQ831520*	a
EU755374	b	FJ660967*	b	GQ915289*	b	HM038026*	b	HQ395019*	b	HQ831521*	b
EU755375	b	FJ660968*	b	GQ995582	b	HM038027*	b	HQ395020*	b	HQ831522*	a
EU755376	b	FJ660970*	b	GU001710*	d	HM038028*	b	HQ395021*	d	HQ831525*	b
EU755377	b	FJ660971*	b	GU049340*	a	HM038029*	b	HQ395022*	d	HQ831526*	b

EU755378	b	FJ667582*	d	GU083582*	b	HM038030*	d	HQ395023*	d	HQ831528*	b
EU755379	b	FJ667583*	d	GU083583*	d	HM038031*	d	HQ395024*	d	HQ831530*	b
EU755380	b	FJ667584*	b	GU124593*	d	HM038033*	a	HQ395025*	d	HQ831531*	b
EU755381	b	FJ667585*	d	GU233804*	b	HM038034*	a	HQ395027*	b	HQ831532*	b
EU780074*	b	FJ667586*	d	GU244506*	b	HM102350*	d	HQ395028*	b	HQ831533*	a

GAN	G	GAN	G	GAN	G	GAN	G
HQ831534*	b	JF927982	b	JN382190	b	JX912914*	d
HQ831535*	b	JF927983	b	JN382191	b	JX912915*	d
HQ831536*	a	JF927984	d	JN382192	b	JX945575*	b
HQ831537*	b	JF927985	d	JN411094	d	JX945576*	b
HQ831538*	b	JF927986	d	JN411095	d	JX945577*	b
HQ831539*	a	JF927988	d	JN411096	d	JX948769*	d
HQ831540*	b	JF927989	d	JN411099	d	JX948770*	d
JF272497*	b	JF927990	b	JN411100	d	JX948771*	d
JF272498*	d	JF928002*	d	JN615187*	d	JX948775*	d
JF272499*	b	JF928004*	d	JN644769	b	JX948776*	d
JF317565*	b	JF928005*	b	JN644770	b	JX948777*	d
JF317566*	b	JF928006*	d	JN644771	b	JX948778*	d
JF317568*	b	JN006445	d	JN660055*	d	JX948781*	d
JF317571*	b	JN006448	d	JN662673	b	JX948782*	d
JF317573*	b	JN006451	b	JN662675	b	JX948783*	d
JF317575*	b	JN006452	b	JN662684	b	JX948784*	b
JF317577*	b	JN006453	b	JN989553	b	JX948785*	d
JF317578*	b	JN006454	b	JN989554	b	JX948786*	d
JF317583*	b	JN006455	b	JN989555	b	JX982219*	d
JF317584*	a	JN006456	b	JN989556	b	JX982220*	d
JF317586*	d	JN006457	b	JN989557	b	JX982221*	d
JF317588*	b	JN006458	b	JQ002671*	d	JX982222*	d
JF682791*	a	JN006462	b	JQ002672*	b	JX982224*	d
JF682792*	b	JN006463	b	JQ181585*	b	JX982226*	d
JF682793*	a	JN006464	b	JQ181589*	b	JX982227*	d
JF682794*	a	JN006465	a	JQ181600*	d	JX982228*	d
JF683387	a	JN119255*	d	JQ387582	b	KC153106*	d
JF683388	a	JN119256*	b	JQ390467*	d	KC188796*	a
JF683389	d	JN119257	d	JQ413808*	d	KC249977*	d
JF683391	a	JN133304*	a	JQ653449*	d	KC447454*	d
JF683392	d	JN133305	a	JQ809463*	d	KC473166*	d
JF683394	d	JN176181*	d	JQ809464*	d	KC473167*	d
JF683398	a	JN382157	a	JQ866914	b	KC473168*	d
JF683399	d	JN382159	b	JQ866915	b	KC527542*	d
JF683400	b	JN382161	a	JQ866918	b	KC533811*	d
JF683401	d	JN382162	a	JQ866919	b	KC533812*	d
JF683402	d	JN382164	b	JQ994269*	a	KC618389*	a
JF683403	a	JN382165	b	JX204386*	d	KC800637*	d
JF683404	d	JN382166	b	JX406419*	d	KC800638*	d
JF683405	d	JN382168	b	JX406420*	d	KC800639*	d
JF683406	b	JN382169	b	JX406421*	d	KC800640*	d
JF683408	d	JN382171	b	JX406422*	d	KC800641*	d
JF690916*	b	JN382172	b	JX406425	d	KC800642*	d
JF690917*	b	JN382173	b	JX512854*	a	KC800643*	d

JF690919*	b	JN382174	b	JX512858*	b	KC800644*	d
JF690920*	b	JN382175	b	JX512859*	b	KC800645*	d
JF718784*	d	JN382176	b	JX512860*	b	KC800646*	d
JF899334*	d	JN382177	a	JX534236*	b	KC835190*	b
JF927976	b	JN382180	b	JX534237*	b	KC835192*	b
JF927978	a	JN382184	b	JX535296*	d	KC835193*	b
JF927979	a	JN382185	b	JX535297*	d	KC860786*	d
JF927980	b	JN382186	b	JX678978*	b		
JF927981	b	JN382188	d	JX679498*	d		

Table S2. Structural analysis by online TopSearch software. The S score corresponds to the number of residues that have structural similarity between each alignment. S_q: indicates the percentage of homologous residues of the structure 3r0r with each of the alignments. S_t: indicates the percentage of homologous residues of each structural homolog aligned with pdb: 3r0r.

PDB ID	Alignment size	S	S_q	S_t
3r0r	194	194	100	100
1c8n	144	115.4	59.5	52.7
1aym	114	93.6	48.2	11.6
1f2n	136	114.4	59.0	19.4

```
                          10        20        30        40        50        60
                  ....*....|....*....|....*....|....*....|....*....|....*....|
gi 340707970  37  NGIFNTRLSRTFGYTIKRTTvktPSWAVDMMRFNINDFLPpgggsnprsVPFEYY-RIRK 95
1AYM 1        67  vesFLGRSGCIHESVLDIVDn-yNDQSFTKWNINLQEMAQirr----kfEMFTYA-RFDS 120
1C8N C        86  GNSTVVSNSELILNLTPi----aLAYTVQSLPLIATQPawlg----tiaDNYSKW-RWVS 136
1F2N C        52  SNTWPLHSVEFLADfkrs----stsADATTYDCVPFNLprvw-----slarCYSMwKPTR 102

                          70        80        90       100       110       120
                  ....*....|....*....|....*....|....*....|....*....|....*....|
gi 340707970  96  VKVEFWPCSPIT-QGDRGVGSSAVILDDNFVTKATALtyDPYVNYSSRHTITQPFS---- 150
1AYM 1        121 EITMVPSVAAKDgHIGHIVMQYMYVPPGAPIPTTRDDy-AWQSGTNASVFWQH-GQ---- 174
1C8N C        137 LRIIYSPKCPTT-T--SGTVAMCLSYDRNDVAPGs---rVQLSQTYKAINFPPYAGydga 190
1F2N C        103 WDVVYLPEVSAT-V--AGSIEMCFLYDYADTIPRY----TGKMSRTAGFVTSSVWY---- 151

                         130       140       150       160       170       180
                  ....*....|....*....|....*....|....*....|....*....|....*....|
gi 340707970  151 -----------Y---------------HSRYFTPKPVLdstidyfqpnnkrnQLWLRLQT 184
1AYM 1        175 -----------P---------------FPRFSLPFLSI--------------ASAYYMFY 194
1C8N C        191 ailntdvtptsA---------------IYVDVDVTRFD--------------KAWYStig 221
1F2N C        152 -----------GaegchllsggsarnaVVASMDCsr---------------vGWKRvtss 185

                         190       200       210       220       230
                  ....*....|....*....|....*....|....*....|....*....|....*....
gi 340707970  185 a--------------GNVDHVGLGTAF-ENSI---YDQE-YNIRVTMYVQ--F-REFNL 221
1AYM 1        195 dgydgdtyksrygtvVTNDMGTLCSRI-VTSEqlhKVKV-VTRIYHKAKHtkA-WCPRP 250
1C8N C        222 taafaa---ltafdqnqFCPCTVHIGS-DGGPa-vAVPP-GDIFFKYVIE--L-IEPIN 271
1F2N C        186 ipss-----vdpnvvntILPARLAVRSsIKPT---VSDTpGKLYVIASMV--LrDPVDp 234
```

Figure S2: Sequences alignment of secondary structure information. Alignment between 3r0r and 1aym, 1c8n and 1f2n proteins. The light gray residues were eliminated from the alignment.

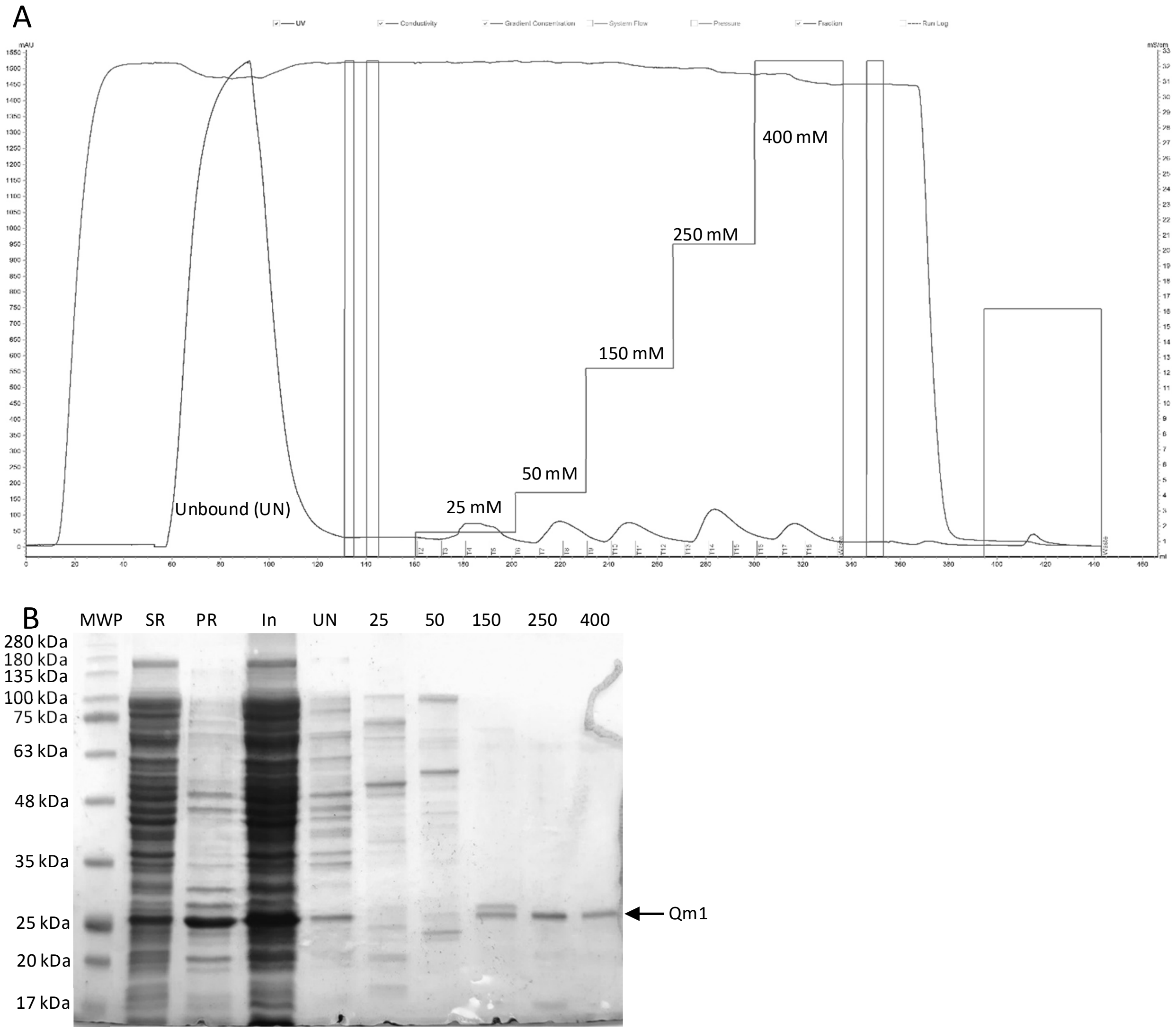

Figure S3. Qm1 purification by IMAC. **A)** Chromatogram resulting from the IMAC Qm1 purification. UN: unbound proteins, 25, 50, 150, 250 and 400 mM correspond to the imidazole concentration in the elution buffer respectively. **B)** SDS-PAGE Coomassie blue staining on reducing condition MWP: molecular weight pattern; SR: supernatant of rupture; PR: pellet of rupture; In: initial sample; UN: unbound protein sample; 25, 50, 150, 250 and 400: samples obtained from IMAC purification. 20 µL per sample was used in each case in both conditions.

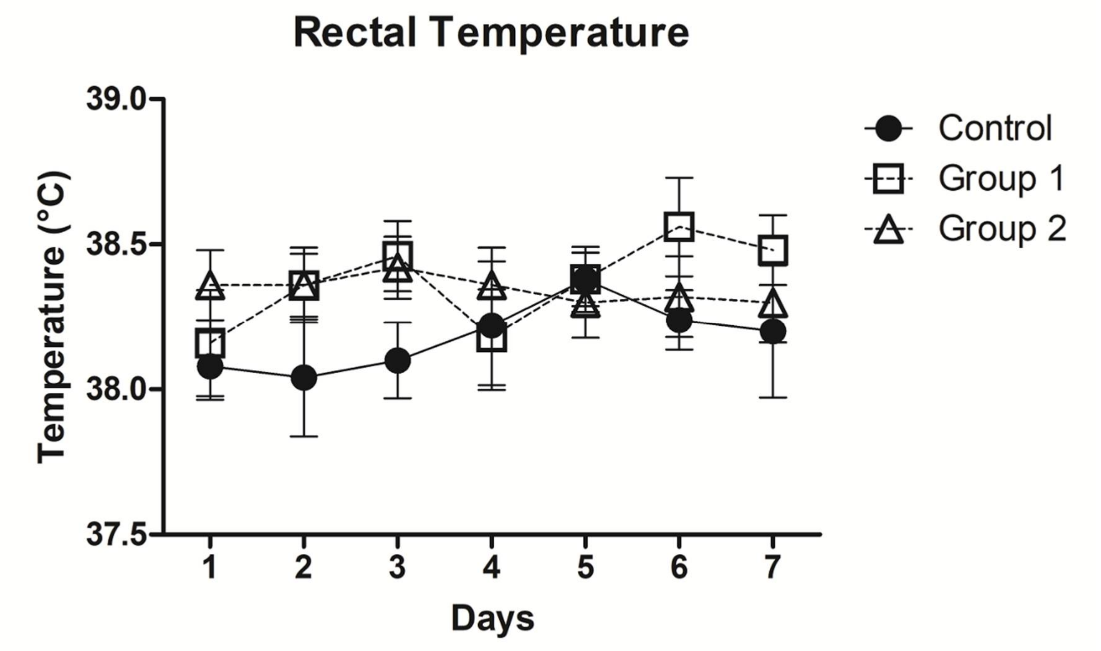

Figure S4. Rectal temperature assay. The rectal temperature was measured daily for 7 days. Values are mean ± SEM. n=5, Two-way ANOVA and Sidak test without significance different versus control group.

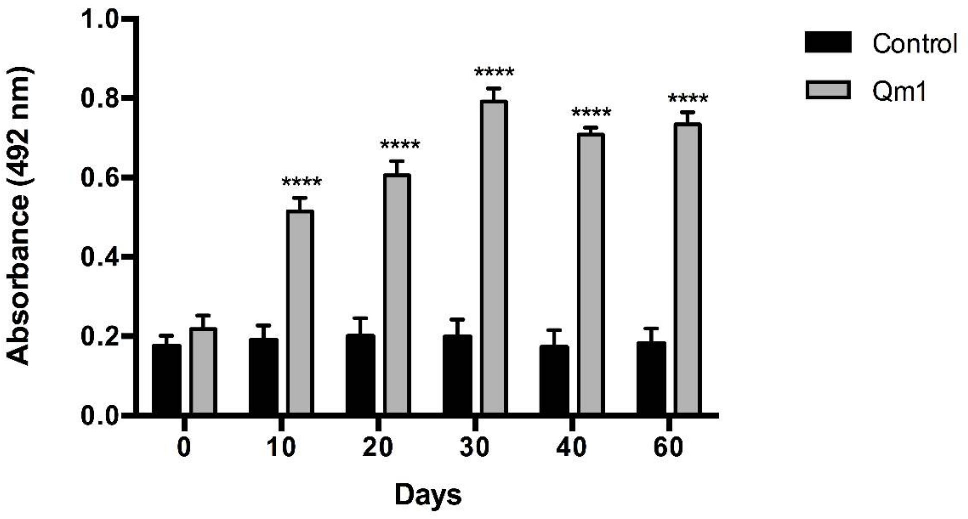

Figure S5. Response to Qm1 vaccination in pigs. Total IgG was measured by indirect ELISA, using serum diluted 1:1000 at the indicated days. Values are mean ± SEM. n=6, Two-way ANOVA and Sidak test ****p<0.0001 versus the control group
